# Science in Medicine and Surgery

**Published:** 1893-05

**Authors:** J. McFadden Gaston

**Affiliations:** Atlanta, Ga.


### SCIENCE IN MEDICINE AND SURGERY.*

By J. McFADDEN GASTON, M. D.,
ATLANTA, GA.

In the early history of the healing art, all the devices used were of an empirical nature, not even suggesting any kind of theory or explanation upon which a resort to treatment was based.

Old records touching upon the various medical and surgical appliances seem so absurd that it puzzles any intelligent physician of the present day to believe that a sane man or woman could have fallen into the delusion to adopt such means of treatment. It is

*Read at Americus before the Medical Association of Georgia, April 19, 1893.

not the purpose of this paper to convict the originators of such means as impostors, nor their victims as fools, but simply to adduce the facts in verification of the extreme gullibility of the human race, as to curative agents. This holds not alone in respect to the past, but also in regard to the present era, and has facilitated the sharp practice of quacks in all ages of the world.

After medicine and surgery emerged from the period in which sorcery and witchcraft dominated the people, there commenced a gradual development of measures founded upon the observation of facts, and the record of experience became the standard for the guidance of practitioners. What proved to give relief in given conditions was regarded as applicable in similar cases, and thus a long array of data was accumulated upon which the *materia medica* has been established under the various classifications of purgatives, emetics, etc.

After the discovery of the circulation of the blood by Harvey, surgery was no longer working blindly as before, and surgical pathology has steadily gained ground, while operative measures have made remarkable advances since that time.

The germ theory of disease has wrought a revolution in the views and practice of physicians and surgeons during late years, which bids fair to place medicine and surgery upon a scientific basis. While there is a vast difference in the opinions of prominent men in the medical profession as to the bacterial origin of disorders, the investigations in progress must ere long culminate in a final adjustment of this question.

That micrococci are associated with the progress of diseases, involving the different structures, has been demonstrated clearly by the microscope; and experiments tend to prove that certain disorders are propagated from one person to another by means of such germs. The modes adopted for testing the introduction of microbes into the system have not afforded any conclusive evidence as to the etiological factor in the development of morbid products. But the observation of bacteriologists has gone very far towards establishing the existence of special forms of bacilli in different types of disease. Whether these various modifications of the bacterial order enter as a causative element, or simply as a concomitant of the several disorders with which they are associated, has not been satisfactorily elucidated.

If the investigations should be conclusive in regard to the constant appearance of distinct bacilli in a large proportion of diseases, so as to serve the purpose of diagnosis, it would conduce greatly towards placing medicine and surgery upon a scientific basis. This being attained, the next step to be aimed at is the therapeutic measure, which may prove efficacious in eliminating these morbific agents from the different structures of the body and correcting their ravages in the organs.

A grave point for consideration in connection with the development of bacteria in the physical organization is whether they are hurtful in the living condition or after losing their vitality and acting as ptomaines in the organs. If the analogy of the growth and the decay of hydatids in the tissues can hold in the case of bacteria, it may be inferred that the chief harm results from their death and decomposition.

A very interesting view of the relation of leucocytes to bacteria has been presented by Sutton, claiming that an interminable war is carried on between these infinitesimal antagonists in the organization. If the former prevail health is secured, but in case the latter exert a controlling influence, the result is disease. The object of medical science is to aid the leucocytes.

At this time the medical profession is undergoing a most interesting transition from the extreme views which have been held by some in regard to the employment of germicides in surgical practice. There was a time within the past decade when it was deemed to be scientific and progressive to use antiseptic measures of the most energetic kind in all operations, whether there was a septic element to combat or not. But thanks to the mature investigation of the effects of germicides upon normal structures by bacteriologists of the highest order of qualifications for this class of work, it has been demonstrated that these so-called antiseptic agents are capable of setting up septic processes in healthy tissues. The tables are now turned, and instead of a surgeon being compromsied by eliminating germicides from his surgical procedures in ordinary cases, and confining his irrigation of recent wounds to simple sterilized water, it is he who departs from this course by the employment of solutions impregnated with toxic agents who is held responsible for the consequences of their absorption. There was a time, and that recently, when prominent members of the profession would have gone into a court of justice and testified that another physician or surgeon who had failed to use the so-called antiseptic treatment in a given case had failed to do his duty, if not even liable to a charge of malpractice. But let us consider where this conservative practitioner now stands with the development of science in the researches of bacteriology. When there is a septic contamination in a part, germicides are indicated for the correction of this vitiated condition and should be carried no further than requisite for this purpose. But in case there is a healthy and normal state of structures involved in a cutting operation, there can be no indication for antiseptics and their use is only calculated to do harm by absorption, and the best men in the profession have ceased to employ them.

The most energetic, and hence the most likely to cause mischief, is corrosive sublimate. In a paper by Dr. A. Morgan Vance, of Louisville, in the *Medical and Surgical Reporter*, it is stated that three years ago Welch, of the Johns Hopkins University, made a report in which he mentioned numerous experiments with sublimate to find out something about the action and power of this drug. "I think," says Dr. Vance, "it is proven beyond all doubt by his experiments and by those of his attendants, particularly Abbott, that the present position of bichloride is rather against its use, and that it does more harm than good. If the solution be of sufficient strength to have any effect upon germs, already there, it will cause necrosis and prevent healing of the wound as well as produce a better nidus for the production and operation of the micro-organisms." In the light of these experiments and those of the experiments quoted in this paper, it is plain that for use in surgical practice the solutions of corrosive sublimate do not possess the advantages hitherto attributed to them. I may be allowed to refer those who still cling to this idol to the practical remarks of Dr. Vance on this subject, as follows: "Two years ago I ceased the use of solutions, particularly in fresh wounds, and have lately ceased to use them at all, even in suppurating conditions. I have found in my practical work that since I ceased the use of sublimate solutions I have had much more uniformly good results by using simply sterilized water."

In my address at the opening of the session of the Southern Medical College in 1888 I made my position clear upon this subject in the following words:

"With the lights before the profession in regard to the cases of poisoning resulting from even the most cautious employment of recognized dilutions of both carbolic acid and bichloride of mercury in surgical dressings, I am forcibly impressed with the conviction that they should be discarded in all operations upon normal structures." In the discussion upon aseptic and antiseptic surgery before this Medical Association in 1890 I used this language: "The excessive use of carbolic acid and corrosive sublimate as washes to absorbent surfaces cannot be otherwise than hurtful; and the practical results in setting up disorders of various kinds, with not a few fatal results, should be a warning to surgeons against the unnecessary employment of these agents. With proper safeguards in the use of antiseptics surgery will reap benefits; but it has received a black eye from extremists, which can only be remedied by caution in the employment of septic agencies, and seeing the danger, let us avoid it. If we have it in our power to employ antiseptic agents free from risk, it is not good surgery to rush to germicides which are found prejudicial to the otherwise healthy structures involved in an operation; and it is high time that the voice of the profession should be raised in tones which cannot be misapprehended against this reckless employment of septic germicides."

I am happy to record that now the progressive element in surgery has adopted the standard under which I have battled for many years past. Many will doubtless continue for a time to irrigate fresh cut surfaces with solutions of corrosive sublimate, even in the face of a scientific demonstration of their detrimental effects. But this surgical fad cannot any longer exert its sway over the thinking men in the profession, and like the carbolic spray of quondam repute, corrosive sublimate washes will ere long be among the things that were, a schoolboy's tale, the wonder of an hour.

It may, peradventure, appear to some of my colleagues that I am falling into line with the reform which has been brought about latterly in cutting loose from the prevailing practice of the last decade; but fortunately I am on record in the plainest and most unmistakable terms during all these years as opposing this class of so-called antiseptic surgery in the indiscriminate employment of sublimate solutions in operations. While I have been surrounded with colleagues who insisted upon carrying out the measures inculcated by the extremists in this antiseptic craze, I have at all times and under all circumstances kept up a defensive warfare on the line of conservatism. They will all do me the justice to allow that there has never been any concealment of my views, and even at the risk of prejudice to my professional prestige in the college with which I have been associated, my position has been boldly and openly asserted in the lecture room and elsewhere as inimical to the septic consequences of washes and dressings of fresh wounds with corrosive sublimate solutions.

A theory based upon the claim that all diseases have their origin in the introduction of germs of one or another order may prove the most satisfactory explanation of the disorders of the physical organization, and should it be made apparent that every special disease has its specific bacillus, science will have made an immense stride in pathology. But when the microscope and other means of investigation shall have demonstrated this as a fact, the more practical and useful discovery of remedies adequate to the destruction or elimination of these bacteria appeals with urgency to the therapeutist; and thus far the bacteriologists have signally failed to point out the curative processes, which may meet the requirements. Much is expected from scientific bacteriology and for one I am anxiously looking for a solutiou of the problem of curing disease by germicides, in some way, which shall not interfere with the vital functions of organs or tissues involved.

In the meantime we must not ignore the time honored principles which have brought us safely thus far in arresting the progress of local and general derangement of the system. Prophylaxis is more important than therapeusis; and the old hackneyed phrase—that an ounce of preventive is worth more than a pound of cure, exemplifies this fundamental doctrine. Jenner conferred the greatest boon upon mankind in the discovery of vaccination as a security against smallpox. Pasteur accomplished a result of no less moment in developing a process of attenuation in the anthrax virus, which put a stop to the ravages of splenic fever. He has in like manner succeeded in so modifying the virus of rabies as to render the canine race insusceptible to this scourge. As a corollary of this demonstration, it has been held that even the human being may be treated during the incubating period of hydrophobia by a gradual introduction of the attenuated virus so as to protect the person from the development of this dreadful disease. More recently an innocent virus obtained by the action of gastric juice upon the spinal cords of rabbits, infected with rabies, has been devised by Eusebio Valli, an Italian physician, and utilized by Centanni as an antidote to hydrophobia by inoculation. Even the wide-spread fatality of yellow fever has been limited materially in its destructive effects by inoculation with attenuated virus in South America by Dr. Domingos Freire of Rio de Janeiro.

The outcome of this procedure for mitigating if not preventing entirely the progress of the yellow fever when it prevails is of highest consequence to our southern population. I have been observing this work, as reported through the newspapers of Brazil, and feel confident that it is destined to bring about important results wherever the yellow fever inoculation is adopted. Out of 11,881 inoculations made by Freire in Brazil, there have only been reported a very insignificant number of deaths, the general mortality being five tenths of one per cent. of those who were inoculated. In the meantime the fatality of those not inoculated has been very great

A sad and striking contrast has been presented among our own people in Brazil by the death of five missionaries from the yellow fever who had not availed themselves of the protective inoculation. In the meantime, one who was inoculated, and gave to the world a report of its characteristic influence upon him, has escaped, though frequently exposed to epidemics of yellow fever.

An effort was made some years ago to have a commission of three competent persons sent to Rio de Janeiro by the Government of the United States to report upon the facts connected with yellow fever inoculation. But after the favorable action of the American

Medical Association, requesting the President to send this commission, Dr. J. B. Hamilton, of Washington city, succeeded, by special parliamentary tactics, in having the action of that body rescinded. Dr. George Sternberg, of the United States Army, in the meantime was sent to Rio de Janeiro and, as was anticipated, brought back a report adverse to inoculation.

The most notable feature of the past and present status of surgery is the independent attitude of the great mass of the profession in saying and doing what each individual thinks best, having no regard for the dictum of authorities, as in former days. We may arrive at a correct idea of the status of surgery then and now by consideration of professors in schools of former days as compared with recognition of others at the present day. A review of the surgery of the brain, thorax, abdomen, rectum, genito-urinary organs, blood vessels, nerves, spine, hip joint, knee and feet of the past with practice at this time, shows vast improvement in all the means of treatment.

It is proper in this connection to advert to the vast increase of facilities for instruction offered by post graduate schools, which were not available prior to the last quarter of a century. If this work was entrusted to experienced men in the different departments, it ought to prove most serviceable to those entering upon the practice of medicine or surgery.

